# Standardized suturing can prevent slackening or bursting suture lines in midline abdominal incisions and defects

**DOI:** 10.1007/s10029-022-02659-x

**Published:** 2022-08-23

**Authors:** C. Lesch, K. Uhr, M. Vollmer, R. Raschidi, R. Nessel, F. Kallinowski

**Affiliations:** 1 General, Visceral and Transplantation Surgery, University Hospital Heidelberg, Im Neuenheimer Feld 410, 69120 Heidelberg, Germany; 2 Hamburg University of Technology, Biomechanics, Denickestrasse 15, 21073 Hamburg, Germany; 3 Department Allgemein- Und Viszeralchirurgie, Spital Walenstadt, St. Gallen, Switzerland; 4 General, Visceral and Pediatric Surgery, Klinikum Am Gesundbrunnen, Am Gesundbrunnen 20-26, 74078 Heilbronn, Germany

**Keywords:** Suture standardization, Incisional hernia, Suturing technique, Slackening suture line, GRIP, CRIP

## Abstract

**Purpose:**

Incisional hernias often follow open abdominal surgery. A small-stitch–small-bite suture might close the incision durably. We analyzed specific details of this closure technique and assessed their influence on the closure stability.

**Methods:**

The effects of cyclic loads, simulating coughs were investigated on a bench test. We prepared porcine bellies in the median line and bovine flanks parallel to the muscle fibers with 15 cm long incisions. Then we punched round or rhomboid defects with a diameter of 5–10 cm into the center of the incision. Monomax® 2–0 and Maxon® 1 and 2–0 were used as suture materials. We tested the durability of the closure with pressure impacts of 210 mmHg repeated 425 times. Throughout the experiments, we modified the suturing technique, the surgeon, the tissue tension, the defect size and shape and the suture diameter.

**Results:**

Standardizing the suture technique improved the durability of the closure significantly. Any other variations showed minor influences after standardization. All incisions with round defects up to 7.5 cm width withstood 425 impacts using standardized suturing. Unstandardized sutures failed in all cases. When closing an incision with a 10 cm wide defect, the tissues ruptured frequently next to the suture line. We defined criteria to standardize this suturing technique. For the first time, we developed a suture factor related to the durability of a sutured tissue closure. We integrated the suture factor into the concept of biomechanically durable repairs.

**Conclusions:**

Suturing the abdominal wall with a standardized suturing technique improves its durability significantly.

## Introduction

Durable closure techniques should be tested in a lifelike experimental setting, simulating everyday activities. Deriving from material science, it is necessary to investigate closure materials with tissues in a compound. We developed a bench test to fulfill these criteria. It allows the study of a repair under cyclic, repetitive loads [1]. The biomechanical properties determine the durability of a repair. These properties include the dynamic stiction of the materials, the closure technique and the tissue qualities of the individual [2, 3].

Repeated increase of the intra-abdominal pressure stresses the abdominal wall. An existing abdominal wall defect gets loaded simultaneously [4]. High peak pressures occur during everyday activities, such as sports or coughing. Pressures of more than 200 mmHg can develop for less than 1 s [4, 5]. One third of our patients cough more than 400 times in the first 24 h postoperatively [6]. Such inevitable loads increase the risk of a failed closure [7].

A hernia occurs when the defect closure can no longer withstand the stress [8, 9]. The failure starts by slackening of the suture line or by tearing of a stitch through the tissue [10]. Suture slackening usually begins early after closure [11, 12]. The resulting fascial dehiscence is mostly invisible. Herniation usually follows a fascial dehiscence that expands to more than 11 mm in 4 weeks [13]. Proper healing of such a fascial dehiscence is rare, since non-cross-linked collagen has a low retention force [14].

Burst abdomen occurs in 3–5% of abdominal closures [15]. Incisional hernias develop in 10–80% after laparotomy [16]. A suture-to-wound length over 4:1 and the use of the small-stitch–small-bite technique is recommended although it cannot fully prevent burst abdomen or hernia recurrences [15]. The observed variation may be the result of the lack of standardization of the saddles, knots, stitch length, depth and tension, loops and widths of the sutures [17].

The aim of this study was to investigate the influence of technical details of a small-stitch–small-bite [18] suture on its durability. Primary endpoint was the condition of the closure after 425 cyclic loads. We examined the biomechanical properties of the tissues and we varied the repairs. We altered the suturing technique, the defect size and shape and the suture diameter. The study design considered the individuality of the surgeon, the tissue elasticity and tension.

## Materials and methods

### The bench test for dynamic intermittent strain [19]

**Fig. 1:**
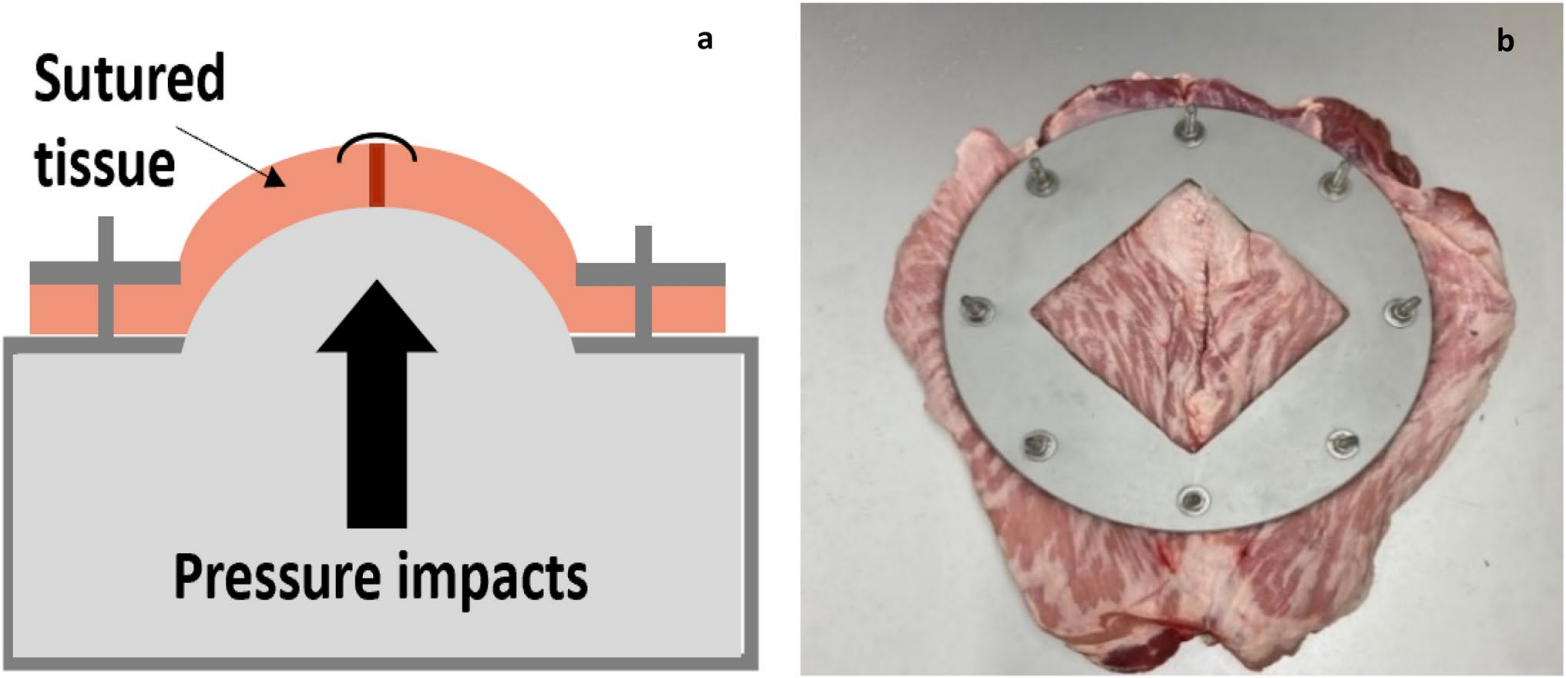
**a** Sketch of the test bench and its function; **b** photograph of sutured porcine tissue on the test bench before cyclic loading; the tissue is held in place with screws tightening the metal ring on the edge of the tissue

We conducted the study with sutured tissues. These tissues were submitted to cyclic load on a self-built bench test [3]. The test bench delivers cyclic pressure impacts with an adjustable pressure plateau phase (Fig. 1). This simulates heavy coughing. The sutured tissue gets loaded 425 times with pressure peaks around 210 mmHg.

**Fig. 2:**
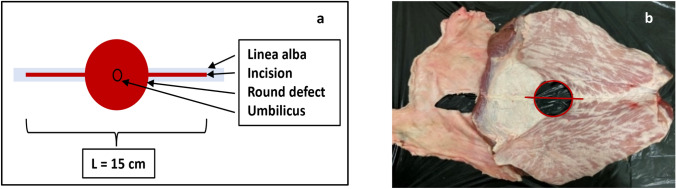
**a** Schematic outline of a prepped round defect (L = length); **b** actual defect in the porcine tissue outlined in red

The study used porcine bellies and bovine flanks as elastic and stiff tissue types [1]. The variation of the tissue elasticity in porcine and bovine tissues is lower than in human tissue with a ratio of 1: 4. However, it covers 95% of the variation found in 123 patients [20]. We cut a median 15 cm long incision into the tissues. Then we punched an additional round or rhomboid defect in the middle of the incision to simulate an abdominal wall defect (Fig. 2). The round defects measured 5 cm, 7.5 cm or 10 cm in diameter, the rhomboid defects were 5 × 15 cm large.

The study design included stiffer porcine or more elastic bovine tissue as well as different defects. We applied various closure techniques, altered tissue tension and three surgeons to assess interobserver variation. The nomination of the parameters is listed in Table 1.

**Table 1:** Nomination system (no add. = no additional abbreviation)

Abbreviation		Meaning
P	=	**P**orcine
B	=	**B**ovine
DR5 × 15	=	Central **D**efect**, r**hombic**, 5 × 15 cm**
DC5	=	Central **D**efect, **c**ircular, **5** cm
DC7.5	=	Central **D**efect, **c**ircular, **7.5** cm
DC10	=	Central **D**efect, **c**ircular, **10** cm
SSSB-u	=	**S**mall-**S**titch–**S**mall-**B**ite suture, ** u**nstandardized
SSSB-s	=	**S**mall-**S**titch–**S**mall-**B**ite suture, ** s**tandardized
LSLB-u	=	**L**arge-**S**titch–**L**arge-**B**ite-suture, unstandardized
hT	=	Sutured tissue is under **h**igher **t**ension
Repr.A	=	**Repr**oduction of the SSSB-s suture by the same surgeon **A**
Repr.B	=	**Repr**oduction by a different surgeon **B**
1 USP	=	Suture with **USP 1**
(no add.)	=	Suture with **USP 2–0**
(no add.)	=	Monomax® suture
MX	=	**M**a**x**on® suture

The closure of the defects was performed in 20 different manners (Table 2). In four series (ES 1–4), rhomboid defects were closed with Maxon® sutures in USP 1 and USP 2–0. In further four experimental series (ES 5, 6, 9 and 10), 5 cm defects were closed with 2–0 MonoMax® sutures in a small-stitch–small-bite (SSSB) or in a large-stitch–large-bite (LSLB) technique. The surgeon applied a suture-to-wound length ratio (SWL) above 4:1 [12, 15]. In two further series (ES 7 and 8), the surgeon used an USP 1 MonoMax® suture [15]. In these series, a stitch deviation of more than 20% was permitted, deriving from the analysis of stable sutures (cf. p. 11). The average bite separation, length and depth, the incorporated suture length and the suture tension were documented for every suture and photographed. The suture tension was evaluated with a spring scale, measuring how many mm one suture loop could be raised with one Newton of traction. Afterwards, we analyzed the durability of the closures, and we recorded the technical details providing a reliable closure of the defects. In an additional ten series (ES 11–20), a more standardized SSSB suturing technique with a stitch deviation of less than 20% was performed. The suture tension was varied in two of these series (ES 13 and 14). A comparison of surgeons was performed in another two of these series (ES 15 and 16). In the last six series (ES 11, 12, 17–10), the diameter of the round defect was increased stepwise from 5 to 7.5 to ten cm in porcine and bovine tissue. Each experimental setup was repeated ten times totalling 200 tests.

**Table 2:** Overview of the experimental series and the likelihood of a secure closure (LOSC) until suture slackening (SS) and tear-out (ST) with standardized (s) or unstandardized (u) large-stitch–large-bite (LSLB) and small-stitch–small-bite (SSSB) technique

Series	Title	Tissue	Defect shape	Defect size (cm)	Suture USP	SSSB/LSLB suture	Standardization	LOSC until SS (%)	LOSC until ST (%)	Suturing technique	Defect shape	Suture diameter & material	Surgeon	Tissue elasticity	Failure progression	Defect size	Large-bite vs. small-bite	Tissue tension
ES 1	P: DR5 × 15 SSSB-u MX	Porcine	Rhomboid	5 × 15	2–0	SSSB	no	n.a	90		x	x					x	
ES 2	P: DR5 × 15 SSSB-u 1USP MX	Porcine	Rhomboid	5 × 15	1	SSSB	no	n.a	60		x	x					x	
ES 3	P: DR5 × 15 LSLB-u MX	Porcine	Rhomboid	5 × 15	2–0	LSLB	no	n.a	100		x	x					x	
ES 4	P: DR5 × 15 LSLB-u 1USP MX	Porcine	Rhomboid	5 × 15	1	LSLB	no	n.a	100			x					x	
ES 5	B: DC5 SSSB-u	Bovine	Circular	5	2–0	SSSB	no	0	30	x		x			x		x	
ES 6	P: DC5 SSSB-u	Porcine	Circular	5	2–0	SSSB	no	0	0	x	x	x			x		x	
ES 7	B: DC5 SSSB-u 1USP	Bovine	Circular	5	1	SSSB	no	0	30			x			x			
ES 8	P: DC5 SSSB-u 1USP	Porcine	Circular	5	1	SSSB	no	0	10		x	x			x			
ES 9	B: DC5 LSLB-u	Bovine	Circular	5	2–0	LSLB	no	0	10						x		x	
ES 10	P: DC5 LSLB-u	Porcine	Circular	5	2–0	LSLB	no	0	30						x		x	
ES 11	B: DC5 SSSB-s	Bovine	Circular	5	2–0	SSSB	yes	70	100	x				x	x	x		x
ES 12	P: DC5 SSSB-s	Porcine	Circular	5	2–0	SSSB	yes	90	100	x				x	x	x		x
ES 13	B: DC5 SSSB-s hT	Bovine	Circular	5	2–0	SSSB	yes	70	80					x	x			x
ES 14	P: DC5 SSSB-s hT	Porcine	Circular	5	2–0	SSSB	yes	100	100					x	x			x
ES 15	P: DC5 SSSB-s Repr.A	Porcine	Circular	5	2–0	SSSB	yes	90	90				x		x			
ES 16	P: DC5 SSSB-s Repr.B	Porcine	Circular	5	2–0	SSSB	yes	50	100				x		x			
ES 17	B: DC7.5 SSSB-s	Bovine	Circular	7.5	2–0	SSSB	yes	80	100						x	x		
ES 18	P: DC7.5 SSSB-s	Porcine	Circular	7.5	2–0	SSSB	yes	100	100						x	x		
ES 19	B: DC10 SSSB-s	Bovine	Circular	10	2–0	SSSB	yes	90	90						x	x		
ES 20	P: DC10 SSSB-s	Porcine	Circular	10	2–0	SSSB	yes	60	60						x	x		
Significant influence										Yes	Yes	No	No	No	No	No	No	No

DC/R: defect circular/rhomboid; hT: high tissue tension; Repr. A/B: reproduction of a suture line by person A or B; n.a.: not available. x indicates the variables tested int the series, the significance niveau relates to *p* < 0.05

The exact parameters applied in each experimental series (ES 1–20) are listed on the left half of Table 2. On the right side of Table 2 the investigated variations for each series are specified. Here, each associated series is marked with an “x”.

**Fig. 3:**
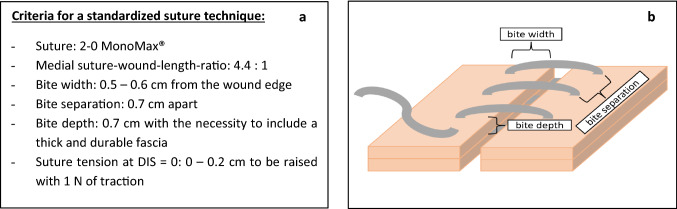
**a** Summary of the criteria for a standardized suturing technique; DIS = dynamic intermittent strain; **b** sketch of the suture and definition of terms

This analysis resulted in the definition of 6 technical details of the SSSB suture (Fig. 3). These details promote a reliable repair through standardization [21, 22]

It is especially important to include a thick and durable fascia in every bite, since the fascia has a higher load bearing capacity than, e.g., muscle only [23]. We chose 7 mm as a standard bite depth, as it promotes that enough durable material is included in the bite.

The test stopped when a failure of the suture line occurred or when 425 DIS impacts were completed. Failure was defined as a slackening of the suture line, the tear out of the suture through the tissue or the rupture of the tissue itself. Slackening means a fascial dehiscence over 5 mm, that is less likely to heal durably. We documented the occurrence and the onset of the failure pattern in the experiments. The study contains a total of twenty series, consisting of 10 experiments each (Table 2).

### Quantification of the influences on the suture line for a reliable defect closure

**Fig. 4:**
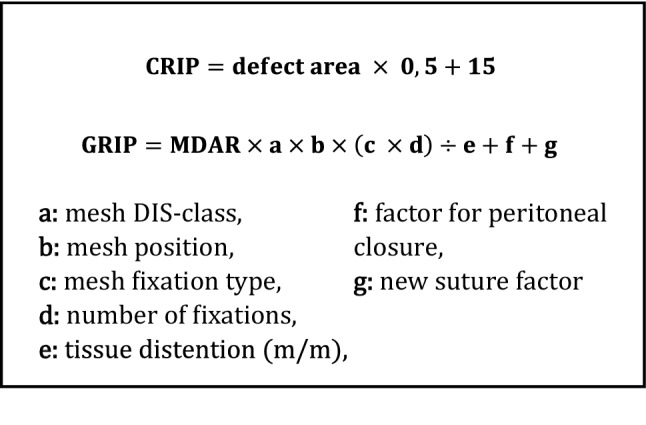
CRIP formula for calculating the necessary stability**.** GRIP formula with factors for calculating the achieved stability**.** MDAR = mesh–defect area ratio [25, 26]. The letters indicate the coefficient for the material and/or technique used

We had the aim to quantify the contribution of a suture line to the overall durability of an abdominal wall repair. We choose the GRIP (gained resistance to impacts related to pressure) concept as a base (Fig. 4). The acronym GRIP describes the gained resistance by the repairs towards pressure impacts [3, 24]. The CRIP (critical resistance to impacts related to pressure) formula assesses the necessary strength as critical resistance to be surpassed for a durable repair [25]. The GRIP formula contains various factors that influence the durability of a customized mesh repair [1, 3]. So far, it was applicable only for unsutured defects and bridged repairs.

We developed a suture factor to integrate suturing for defect closure into the GRIP formula. The factor “g” given above in the GRIP formula obtains an estimate of the durability provided by suturing a defect (Fig. 4). For this purpose, we calculated the CRIP. The CRIP is calculated for the initial defect size according to a previously published formula [25]. For the ease of use, we considered only the round defect area. Yet, in reality the unstable zone of the abdominal wall is much larger in about half of our patients [1, 24]. When GRIP is higher than CRIP, a durable repair is achieved. The newly found suture factor was integrated into the existing concepts. This factor derived from a quantitative and qualitative analysis of the suture’s durability and precision.

### Statistical analysis

Parametric and non-parametric statistical parameters were calculated. Since the results were usually skewed, non-parametric testing was conducted by the Kruskal–Wallis test for the homogeneity of the groups. Differences between two samples were analyzed with a Mann–Whitney *U* test. Box-and-Whisker-Plots and curves reflecting the likelihood of a reliable closure similar to survival curves were used for the visual depiction of the results.

## Results

### Quantification of the influences on the suture line for a reliable defect closure

**Fig. 5:**
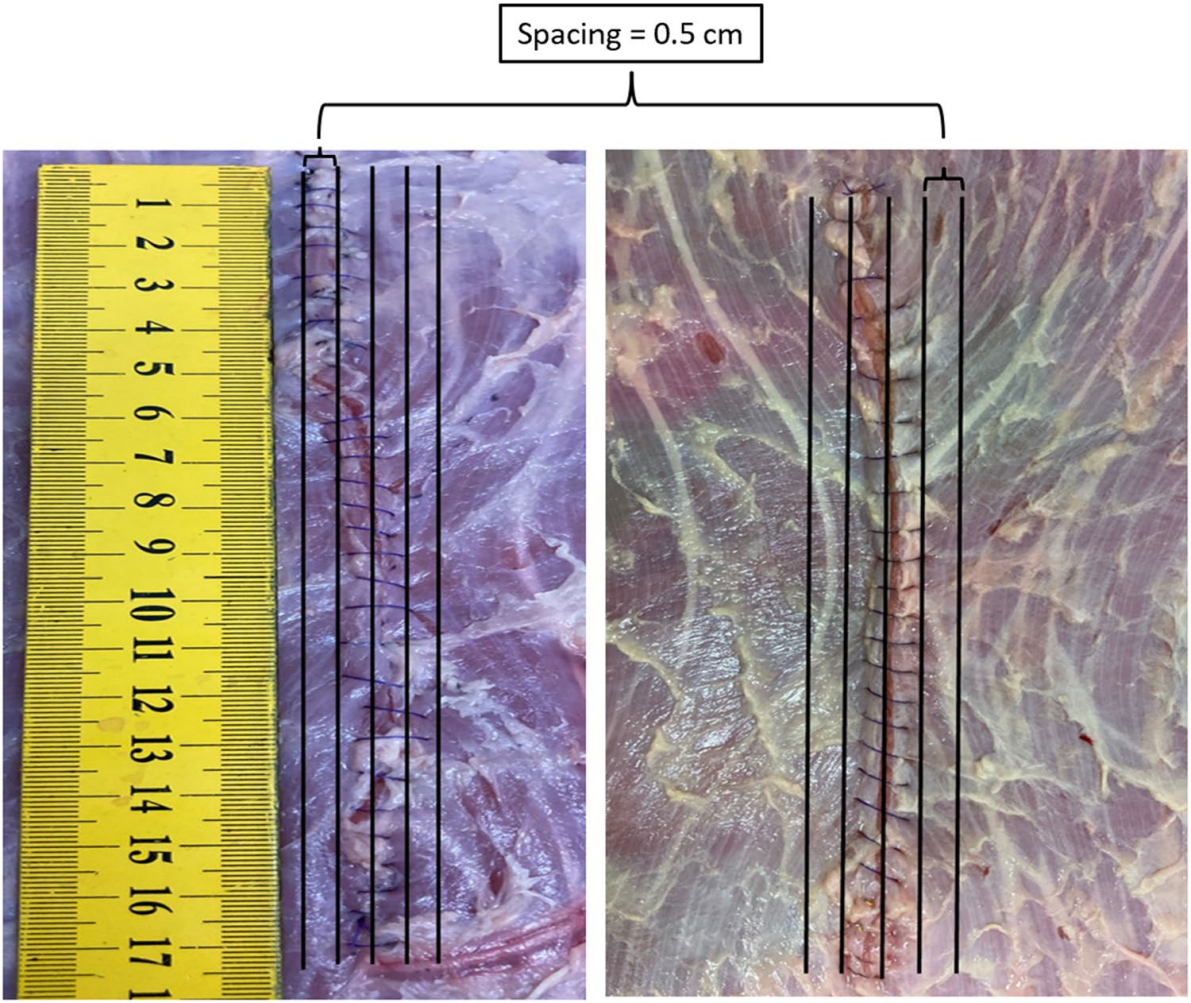
Stitch placement in bovine tissues (left: unstandardized suture; right: standardized suture) with longitudinal evaluation lines to assess the correct position of the stitches. Performing a similar analysis horizontally, the stitch variation exceeds 30% (60–80%) in an unstandardized suture but stays below 20% in the standardized suture

**Fig. 6:**
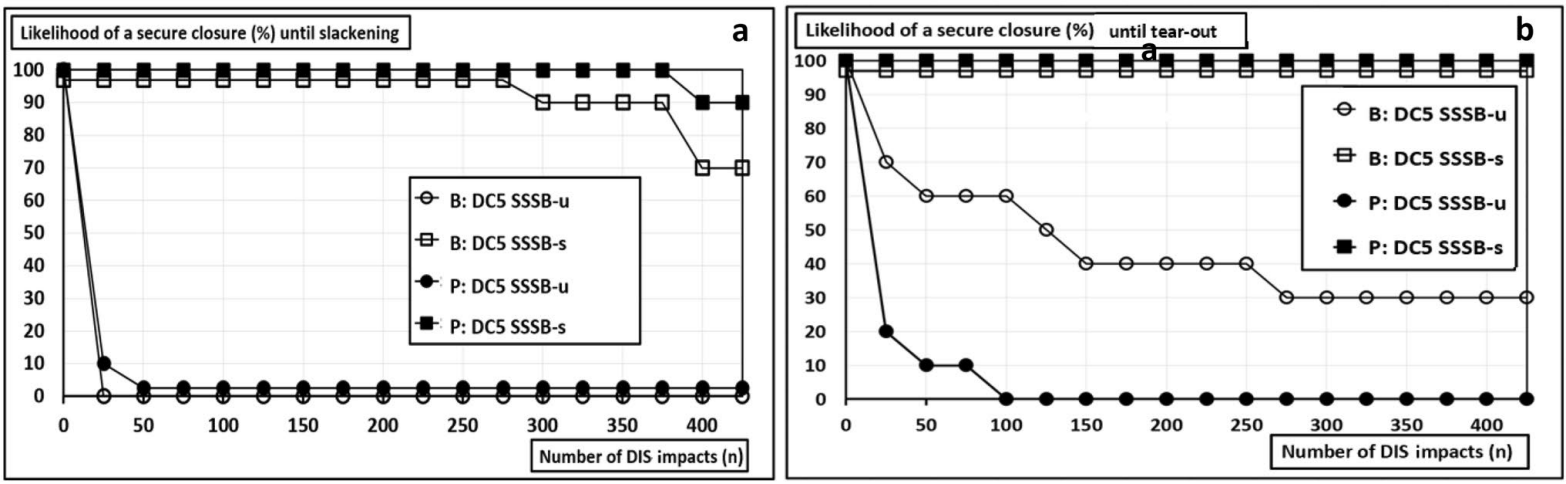
**a** Durability rates until slackening of sutures closing a 5 cm round defect placed centrally in a 15 cm long midline incision in bovine (B) and porcine tissue (P) as a function of the number of DIS impacts in bovine (B) and porcine tissue (P); u = unstandardized, s = standardized suturing; **b** durability rates until tear-out of sutures upon repeated DIS impacts

We found that an unstandardized closure of the fascia in porcine tissue adds a basal suture factor of about 20 to the GRIP. A standardization-specific suture factor adds considerably more to the resistance. To achieve this, the surgeon needs to place every stitch correctly in the horizontal and vertical direction (Figs. 5 and 6). Each stitch can add one additional point neglecting the starting and the closing suture loop with the knot. We analysed the suture lines with the scheme given in Fig. 5. The analysis revealed that durable, standardized sutures exhibit a stitch variation of under 20%. Therefore, the surgeon should place less than 20% of all stitches of a suture imprecisely. A suture with 22 precise stitches can generate a suture factor of 42. In bovine tissue the result is multiplied by 1.5 considering the respective tissue elasticity resulting in a factor of 63. The analysis of an unstandardized and a standardized suture in bovine tissue is visualized in Fig. 5.

### Significant influences

#### Influence of the suturing technique

Standardized sutures closed incisions with 5 cm round defects in all cases. Unstandardized sutures slackened and broke both in the sturdy porcine and in the elastic bovine tissue (Table 2, ES 5 and ES 6).

The standardized sutures were significantly more durable than the unstandardized sutures (Fig. 6, *p* = 0.00008). This applies to both tear-out (B: *p* = 0.00815; P: *p* = 0.00016) and slackening (*p* = 0.00016).

The likelihood of a secure closure measures how many out of 10 sutures (in %) withstood the number of impacts given on the *x*-axis. The two “square” graphs in each panel of Fig. 6 show standardized sutures that provide significantly more stability (*p* values < 0.001). The “circle” two graphs depict unstandardized sutures.

Slackening of the suture line after unstandardized suturing was observed early. All unstandardized sutures slackened beyond the 50th cycle. After standardized suturing, slackening occurred in 10% of the sutures in porcine tissue. 30% of the sutures slackened in bovine tissue starting after 275 impacts (Fig. 6, upper panel). Standardized suturing reached 100% likelihood of a secure closure until tear out (Fig. 6). This applies for both tissue types.

#### Influence of the defect shape

The larger rhomboid defects were easier to close compared to the smaller, 5 cm wide, round defects (SSSB: *p* = 0.00018; LSLB: *p* = 0.00906). Using a continuous unstandardized suture, 60–100% durability could be achieved, depending on the suturing technique and suture diameter. Unstandardized sutures of round defects were durable in 0–30% of the cases (Table 2, ES 1, 2, 3, 6 and 8).

### Insignificant influences

As summarized in Table 2, we modified various parameters throughout the experiments. Only the standardization of the suture and the defect shape show significant differences. None of the other variations lead to significant influences. The results of the different insignificant modifications are described below.

#### Influence of the suture diameter and material

The MonoMax©-2–0 suture did not show a significant advantage over the MonoMax©-1 suture for an unstandardized suture (ST: B: *p* = 0.385; P: *p* = 0.096). The larger suture tended to be more durable in bovine and porcine tissue until suture tear-out. The difference until slackening was significant in bovine tissue (SS: B: *p* = 0.0052; P: *p* = 0.0756).

The use of Maxon®-2–0 and Maxon®-1 sutures exhibited no significant difference (*p* = 0.307). The thinner suture tended to be more durable when a small-stitch–small bite technique is applied.

#### Influence of the surgeon

Surgeon A observed one tear-out without prior suture slackening. In the parallel series, person B observed no tear-outs (Table 2). However, the suture slackened in 5 of 10 experiments of series B. The differences were not significant (SS: *p* = 0.122; ST: *p* = 0.705). The occurrence of slackening varied between the 30th and the 300th DIS impact. Nevertheless, both surgeons achieved a likelihood of a secure closure of 90–100% until suture tear-out using the standardized suture.

#### Influence of the tissue elasticity

*Unstandardized* sutures tore-out significantly less in elastic bovine tissue compared with stiffer porcine tissue (ST: *p* = 0.00516).

*Standardized* sutures in bovine and porcine tissues showed no significant differences (*p* = 0.969). *Elastic tissue* tended to provide a greater durability until tear-out with standardized sutures. Yet, slackening appeared slightly more often (SS: *p* = 0.471). *Stiff tissue* sustained a lower durability until tear-out, but a reduced rate of slackening. The differences between the occurrence of suture slackening and suture tearing tended to be greater in elastic tissue than in stiff tissue (B: *p* = 0.271; P: *p* = 0.727). Large defects tended to be more difficult to close in stiff tissue (SS *p* = 0.711).

#### Influence of the failure pattern and progression

Throughout the experiments, three different failure patterns were visible. First, the slackening of the suture that results in a fascia dehiscence. Second, the tear-out of a previously slackened suture. Third, the rupture of the tissue next to an intact suture line.

For 5 cm wide defects, failure of the repair seemed to be due to malfunctioning sutures. Slackening could act as a predictive risk factor for suture failure by tear-out.

With standardized sutures the suture line stayed intact. The observed failures were due to the rupture of the tissue next to the intact suture line. This shows that the standardized suture is more stable than the tissue. Therefore, the support of the tissue needs to be considered in the repair.

#### Influence of the defect size

**Fig. 7:**
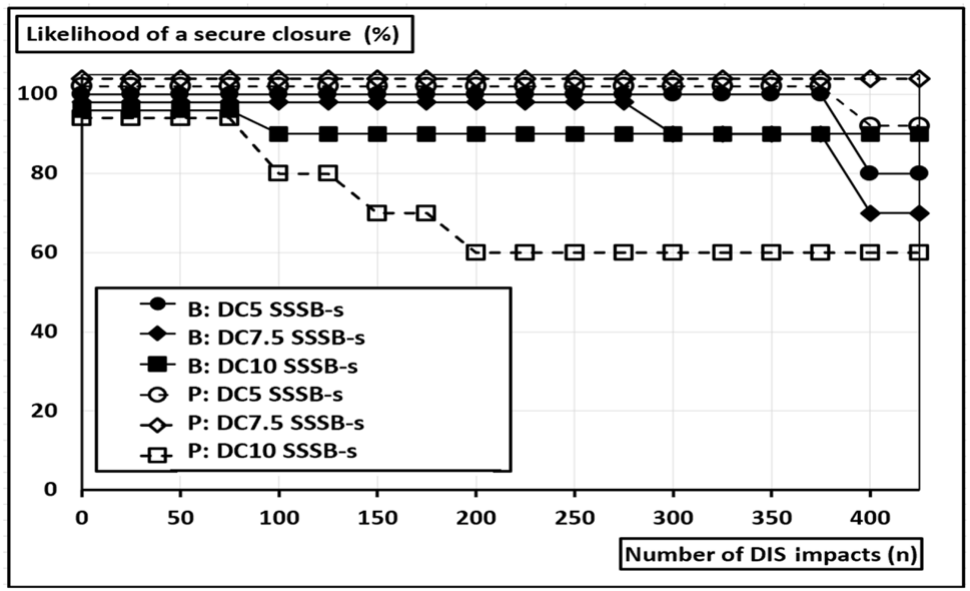
Durability rates until slackening of sutures closing round defects of different diameters in a 15 cm long midline incision in bovine (B) and porcine tissue (P) as a function of the number of dynamic intermittent strain (DIS) impacts in in bovine (B) and porcine tissue (P); u = unstandardized, s = standardized suturing

There was a trend, but not a significant difference in the durability of closed defects with an increasing size up to 10 cm when a standardized suture is applied (SS: B: *p* = 0.803; P: *p* = 0.252) (ST: B: *p* = 0.907; P: *p* = 0.212). As a tendency, the sutures of larger defects show a lower durability, as depicted in Fig. 7.

For *7.5 *cm* wide round defects*, a 100% likelihood of a secure closure was obtained until tear-out of the suture in both tissue types (Table 2). A total of 20% of the sutures in bovine tissue slackened, but none slackened in the porcine tissue (Fig. 6). For *10 *cm* wide, round defects* a 90% likelihood of a secure closure until tear-out was obtained in bovine tissue (Table 3). In the stiffer porcine tissue, the sutures slackened more frequently and provided only 60% durability. Here, all observed suture slackenings resulted in a tear-out.

**Table 3:** Overview of a selection of recently applied hernia models and bench tests

Author	Load delivering method	Load orientation	Disruptiv vs. repetitiv	Used max. load	Number of impacts	Tested components
Siassi et al. 2014 [28]	Hydraulic, cyclic pressure impacts (water)	Multiaxial	Repetitiv	210 mmHg	425	Tissue and Mesh and Suture, staples and/or glue
Sahoo et al. 2015 [29]	Ball-burst-test or traction test	Multiaxial or biaxial	Repetitiv or disruptiv	n.a	1	Mesh
Lyons et al. 2015 [30]	Pressure by air filled balloon	Multiaxial	Disruptiv	Up to 150 mmHg	1	Fixed mesh
Kroese et al. 2016 [31]	Pressure by air filled balloon	Multiaxial	Repetitiv	Average of 75 mmHg		Mesh and suture
Cooney et al. 2018 [32]	Pressure by air filled balloon	Multiaxial	Disruptiv	150 mmHg	1	Tissue and suture

#### Influence of the small-bite- vs. the large-bite-technique

In the conducted experiments, the small-bite technique offered no clear advantage over the large-bite technique. The large-bite suture was found to be significantly more durable in porcine tissue until tear-out (ST: P: *p* = 0.00061). However, until slackening the small-bite suture was significantly more likely to provide a secure repair (SS: B: *p* = 0.00067; P: *p* = 0.00815). Due to these inconclusive results, no clear recommendation can be made whether small-bites or large-bites are more durable.

#### Influence of the tissue tension

An increase of the tissue tension by about 10% lead to an unsignificant 10–20% reduction of the durability (SS: B: *p* = 0.503; P: *p* = 0.968), (ST: B: *p* = 0.912; P: *p* = 0.968) [24].

## Discussion

### Bench test

Different approaches exist to determine the behaviour of sutures and defect closures under load (Table 3). For market certification materials for hernia repair are tested destructively. The influence of the tissue is usually not considered. In contrast, material science tests polymers as composites by applying cyclic loads by default. The test bench used in this work applies cyclic loads to a composite of tissue, suture and textiles. This mimics an abdominal wall setup as a compound. The cyclic loads attain values around 210 mmHg, since intraabdominal pressure peaks up to 280 mmHg occur during daily activities [27].

### Quantification of the influences on the suture line for a reliable defect closure

There is no guideline to determine the optimal closure of an incision or a defect of the abdominal wall. Since 2016, the ratio between the mesh area and the defect area is seen as crucial. This is the base for the concept of mesh–defect-area-ratio, MDAR [26]. A further development of the MDAR concept is the CRIP and GRIP concept (Critical/Gained Resistance to Impacts related to Pressure, Fig. 4) [3, 25]. The GRIP concept considers not only the mesh size but also the mesh type, position, and fixation (cf. p. 6). We based our repairs on these theories. This is the first study assessing the contribution of the suture with an additive suture factor. The retention force of a suture in a specific technique and in tissue with known elasticity is a function of the suture length and its diameter [33]. In clinical practice, a pragmatic approach is needed to assess the quality of the suture line.

Table 4 shows the calculated CRIP and GRIP values as well as the determined suture factors. The gap between the GRIP and CRIP values can be attributed to the strength of the suture. A defect can be sutured durably, and the GRIP value needs to include the contribution of a suture line.

**Table 4:** Overview over different test setups with calculated CRIP and GRIP values and suture factors for standardized and unstandardized sutures (LOSC = likelihood of a secure closure; SS = suture slackening, ST = suture tear-out)

Defect size (in cm)	Tissue	LOSC until SS (%)	LOSC until ST (%)	CRIP circular defect	GRIP	Suture factor	GRIP + suture factor
Unstandardized suture							
5	Bovine	0	30	37.5	0	22.5	22.5
5	Porcine	0	0	25	0	15	15
Standardized suture							
5	Bovine	70	100	37.5	0	63	63
5	Porcine	90	100	25	0	42	42
7.5	Bovine	80	100	56	0	63	63
7.5	Porcine	100	100	37	0	42	42
10	Bovine	90	90	81	0	63	63
10	Porcine	60	60	54	0	42	42

**Fig. 8:**
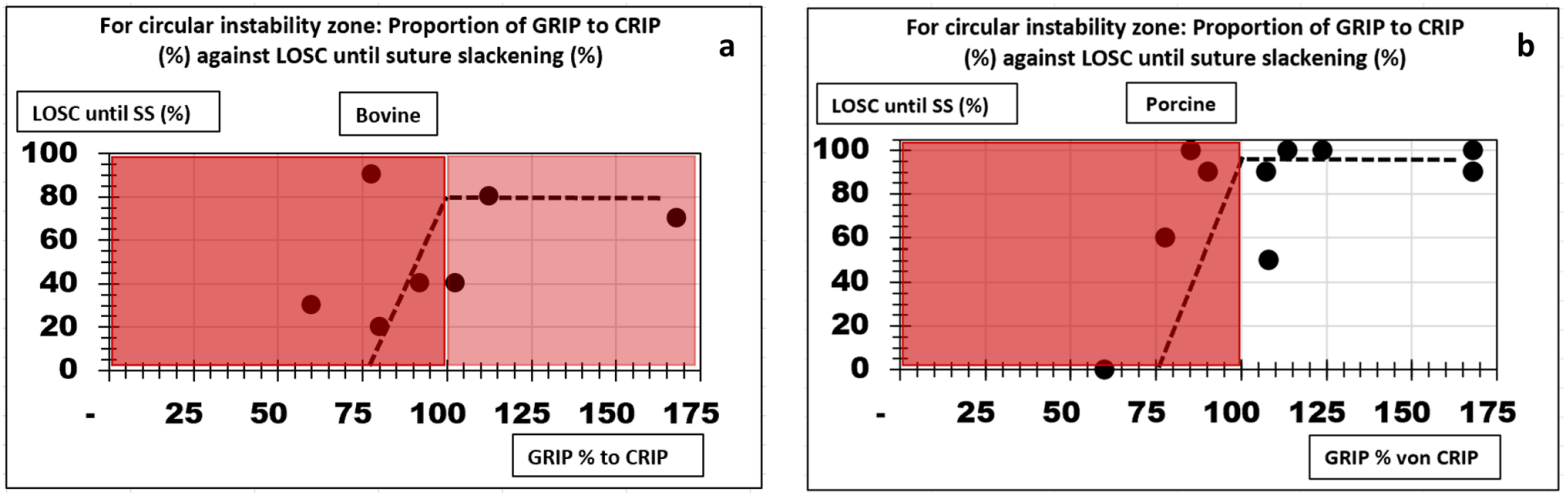
**a** Diagram plots the likelihood of a secure closure (LOSC) until suture slackening (SS) reached with standardized and unstandardized sutures in bovine tissue upon the percentage of the CRIP that is reached by the reconstructions GRIP including the new suture factor. The light red zone indicates that elastic tissue reaches a durability no better than 70–80% and requires prophylactic mesh placements; **b** LOSC until suture slackening of standardized and unstandardized sutures in porcine tissue is plotted against the GRIPs percentage of the CRIP. In both panels, the dark red zone indicates insufficient stability and, therefore, the need of an additional mesh reinforcement

Figure 8 plots the likelihood of secure closure until suture slackening as a function of the percentage of the GRIP and the added suture factor to the CRIP.

The red areas represent the zone in which sufficient durability might not be achieved by the suture alone. A mesh reinforcement of the suture is necessary. The sutures in the more elastic tissue held securely to a maximum of 70–90% only without suture slackening. A mesh reinforcement is always recommended for unstable tissue, as shown in the upper panel of Fig. 8. Mesh reinforcement in stiffer tissue is only necessary when the repair does not reach the CRIP (Fig. 8, lower panel). In further experiments, which were not part of this publication, we found that an additional mesh reinforcement also reduces the risk of suture slackening or facial dehiscence. For 10 cm round defects, the placement of an additional 20 cm round CICAT DynaMesh® mesh without fixation in the sublay position increased the durability from 60% to 90–100% until suture tear-out. A prophylactic mesh reduces suture slackening of unstandardized sutures by 20 to 80%, depending on mesh size and tissue properties.

### Considering the insignificant technical influences

Various parameters can influence the durability of a repair. We distinguished significant and non-significant influences on the durability. The strongest significant improvement was shown to be the standardization of the suturing technique. The defect shape has a significant influence as well. Variations of the suture technique, the suture diameter and material lead to non-significant results. Varying surgeons with the same technique, the defect size, the tissue elasticity and the tissue tension had no significant effect within the limits studied.

According to literature, the suture material only shows slight differences in the sturdiness [34]. The surgeon influenced the rate of suture slackening by 40%. This variation may be due to differences in the application of technical details.

All patients show a variation in the elasticity of their abdominal wall. We conclude that the study of tissues with different tissue elasticities is relevant. The assessment of the tissue properties allows the detection of risks, e.g., by advanced analytical techniques [1]. Such risks can be associated with the different elasticities and the development of specific treatments. There is little knowledge of failure development and progression in tissue–textile compounds. Only the failure prevalence, but not the failure pattern and progression are monitored. Knowledge of the different types of failure and mechanisms could prevent failure early and effectively.

In our study the failures occurred in the middle of the defect line, where the tissue bears the highest load. The first steps of failure like slackening usually occur early after operation [12, 13]. The suture tension seems to be a relevant factor, since a loose suture tends fail faster [35]. An evolving dehiscence might not yet be visible under the skin and subcutaneous tissue. Weak scar tissue is formed, and a secure healing is impaired [9, 10, 14]. In clinical practice, fascia dehiscence greater than 12 mm is followed by hernia formation within 4 years in 94% of cases [10, 13]. To lower the risk of necrosis due to tight sutures, the fascia should be the main load bearing component of the suture. Our choice of an elastic suture material and a continuous suturing technique also lowers the risk for necrosis [36, 37].

In large defects, the suture line stayed intact, but the tissue ruptured parallel to the suture during the bench tests. Consequently, it should not only be the aim to improve the durability of the closure technique but also to consider the strength of the abdominal tissue. An additional prophylactic mesh can support the weak tissues [8]. A tissue fissure occurred relatively more often with standardized sutures.

The results also show that larger round defects tend to be more difficult to repair with sutures only. This is mostly due to tissue failure. Accordingly, a repair that strengthens the tissue is necessary to achieve a secure closure. Fibroblast ingrowth and collagen stiffening requires biomechanical stability. Tissue remodelling under unstable conditions results in elongation of the newly formed collagen and in the formation of an elastic hernia sack [8]. Increasing hernia size is also seen as a risk factor for failure and complications in the literature [38, 39]. The assessment of the hernia size is important for the preoperative planning and for the design of a durable repair with the GRIP/CRIP concept. The surgeon can assess the necessity of a prophylactic mesh preoperatively (Fig. 8).

The validity of the study is limited by the small number of options examined compared to the vast number of technical options, sutures and meshes available. However, an image analysis can possibly form a basis for a prospective comparative study of abdominal closures and prophylactic meshes based on biomechanical aspects. A comparative relative number potentially enables the long-term-strength to be assessed. This has been shown for incisional hernia repair and may be a perspective for abdominal wall closure as well [20]. We are well aware that our studies are only applicable to about 80% of the human cases. However, all examined tissue types can elongate by a median of approx. 20%, so that the elongation behavior of the animal tissues is comparable to human tissue [3]. There might be techniques like the reinforced suture line to strengthen the tissue surrounding the tissue-closing suture [40]. This modification might prevent the tissue failure observed in this study when closing defects with a diameter of 10 cm.

Contrary to current literature, unstandardized sutures had no advantage neither as small-bite sutures nor as large-bite sutures [15, 41, 42]. This expands current knowledge to include biomechanical aspects [18, 43]. A standardization of the suture technique makes a bigger difference. In clinical practice, standardized suturing may be a starting point for future research.

## Conclusions

A standardized suturing technique for the closure of midline incisions and median abdominal wall defects was developed. The standardization improved the durability of a suture repair significantly. After standardization, other variables had little influence on the durability.

The likelihood of securely repairing a 5 cm round defect with unstandardized sutures is close to zero. Therefore, we developed suture parameters to ensure a precisely defined, standardized suture. With this standardized suture technique, we achieved a 100% durability until suture tear-out for 5 and 7.5 cm round defects in 15 cm incisions.

Round defects with a diameter of 10 cm can be closed 90% securely in elastic tissue. Stiff tissue limits the durability at a defect diameter of 10 cm. Accordingly, a prophylactic mesh reinforcement is recommended.

Based on these findings, we quantified the contribution of a suture to the overall durability of a repair for the first time. This newly developed additive suture factor has successfully extended the GRIP/CRIP concept. This work will make it possible to include the individual suture in the preoperative calculation of the stability. In addition, it allows assessing the suture quality intraoperatively.

A standardized suture should be used for all defect closures. An additional prophylactic mesh should be used in high-risk cases, e.g., elastic tissue or defect sizes above 10 cm.
